# Phylogenetics of *Cucumis *(Cucurbitaceae): Cucumber (*C. sativus*) belongs in an Asian/Australian clade far from melon (*C. melo*)

**DOI:** 10.1186/1471-2148-7-58

**Published:** 2007-04-10

**Authors:** Susanne S Renner, Hanno Schaefer, Alexander Kocyan

**Affiliations:** 1 Department of Biology, University of Munich, 80638 Munich, Germany

## Abstract

**Background:**

Melon, *Cucumis melo*, and cucumber, *C. sativus*, are among the most widely cultivated crops worldwide. *Cucumis*, as traditionally conceived, is geographically centered in Africa, with *C. sativus *and *C. hystrix *thought to be the only *Cucumis *species in Asia. This taxonomy forms the basis for all ongoing *Cucumis *breeding and genomics efforts. We tested relationships among *Cucumis *and related genera based on DNA sequences from chloroplast gene, intron, and spacer regions (*rbcL*, *matK*, *rpl20-rps12*, *trnL*, and *trnL-F*), adding nuclear internal transcribed spacer sequences to resolve relationships within *Cucumis*.

**Results:**

Analyses of combined chloroplast sequences (4,375 aligned nucleotides) for 123 of the 130 genera of Cucurbitaceae indicate that the genera *Cucumella*, *Dicaelospermum*, *Mukia*, *Myrmecosicyos*, and *Oreosyce *are embedded within *Cucumis*. Phylogenetic trees from nuclear sequences for these taxa are congruent, and the combined data yield a well-supported phylogeny. The nesting of the five genera in *Cucumis *greatly changes the natural geographic range of the genus, extending it throughout the Malesian region and into Australia. The closest relative of *Cucumis *is *Muellerargia*, with one species in Australia and Indonesia, the other in Madagascar. Cucumber and its sister species, *C. hystrix*, are nested among Australian, Malaysian, and Western Indian species placed in *Mukia *or *Dicaelospermum *and in one case not yet formally described. *Cucumis melo *is sister to this Australian/Asian clade, rather than being close to African species as previously thought. Molecular clocks indicate that the deepest divergences in *Cucumis*, including the split between *C. melo *and its Australian/Asian sister clade, go back to the mid-Eocene.

**Conclusion:**

Based on congruent nuclear and chloroplast phylogenies we conclude that *Cucumis *comprises an old Australian/Asian component that was heretofore unsuspected. *Cucumis sativus *evolved within this Australian/Asian clade and is phylogenetically far more distant from *C. melo *than implied by the current morphological classification.

## Background

Knowing the closest relatives and natural composition of the genus *Cucumis *L. is important because of ongoing efforts by plant breeders worldwide to improve melon (*C. melo*) and cucumber (*C. sativus*) with traits from wild relatives [1]. Next to tomatoes and onion, melon and cucumber may be the most widely cultivated vegetable species in the world [2]. Economic interest from breeders also led to the sequencing of the complete chloroplast genome of *C. sativus *[3]. Evolutionarily, *Cucumis *organellar genomes are unusually labile [4-7], and major chromosome rearrangements are thought to have taken place during the evolution of *Cucumis*. *Cucumis sativus *is the only species in the genus with a chromosome number of n = 7, which is thought to have evolved from a presumed ancestral karyotype with n = 12, but details of this reduction in chromosome number have remained unclear. Thus, the genus *Cucumis *holds great interest as a system in which to study the evolution of organellar and nuclear genomes, and there are also several ongoing efforts to map the genomes of *C. melo *and *C. sativus *[8].

Ongoing work on Cucurbitales and Cucurbitaceae [9,10] has resulted in the generation of sequence data for a dense sample of taxa that together represent 21% of the family's 800 species and 95% of its 130 genera (following the most recent classification, 11]. Early results from this work suggested that *Cucumis *might not be monophyletic. We sought to test the monophyly of *Cucumis *by analyzing a broad sample of taxa based on Kirkbride's biosystematic monograph of the genus [12], other recent studies [10,13], and geographical considerations (independent of traditional assessments of morphology). Robust phylogenetic trees for *Cucumis *might also shed light on the ancestral areas of *C. melo *and *C. sativus*. It is thought that *C. sativus *originated and was domesticated in Asia, while *C. melo *is though to have originated in eastern Africa [14], but with secondary centers of genetic diversity in the Middle East and India [15] and perhaps also China [16]. The center of *Cucumis *evolution is thought to be Africa [12].

The circumscription of *Cucumis *dates back to Linnaeus [17], with the most significant modern change being the separation of *Cucumella *Chiovenda in 1929, which has become generally accepted [11-13,18]. The two genera differ only in the shape of their thecae, those of *Cucumella *being straight or slightly curved, those of *Cucumis *strongly curved and folded. Within the genus *Cucumis*, two subgenera are generally accepted, subgenus *Melo *(30 species, including *C. melo*), with most species in Africa and a chromosome n = 12, and subgenus *Cucumis *(2 species, *C. sativus *and *C. hystrix*), which is confined to Asia and has chromosome numbers of n = 12 and n = 7 [12,19].

Molecular phylogenetic studies of *Cucumis *have sampled up to 16 species of *Cucumis *for chloroplast restriction sites and nuclear isozymes, nuclear ribosomal DNA from the internal transcribed spacer (ITS) region, microsatellite markers, and a combination of RAPDs and chloroplast markers [1,20-22]. With one exception, these studies included only recognized species of *Cucumis*. A further handicap was that the sister group of *Cucumis *was unknown, so that trees could not be rooted reliably. Only Garcia-Mas et al. [22] sampled a potential relative, *Oreosyce africana*, material of which they received under the name *Cucumis membranifolius *Hook. f. and found embedded among species of *Cucumis *(see *Results and Discussion *for a problem with the identification of this material). Morphological similarities, however, argue for adding more representatives from African and Asian genera to phylogenetic analyses of *Cucumis*. Besides *Cucumella*, *Dicaelospermum *C. B. Clarke, *Mukia *Arn., *Muellerargia *Cogn., *Myrmecosicyos *C. Jeffrey, and *Oreosyce *Hook. all share key traits with *Cucumis *[summarized in [23]]. The most recent morphology-based classification of Cucurbitaceae [11] includes five more genera in the tribe Cucumerinae, to which *Cucumis *belongs. No representatives of Cucumerinae were included in previous molecular studies of *Cucumis*.

Because of the doubtful morphological separation from its supposed closest relative, *Cucumella *[12,13,18], and the insufficient sampling of other potentially related genera, the status of *Cucumis *as a monophyletic genus has remained equivocal. Here we address the three questions, Is *Cucumis *monophyletic? What is the closest relative of *Cucumis*? And what are the closest relatives of cucumber and melon?, using a two-pronged approach that involves chloroplast sequence data for all relevant genera of Cucurbitaceae and combined nuclear and chloroplast data for species of *Cucumis*, *Cucumella*, *Dicaelospermum*, *Mukia*, *Muellerargia*, *Myrmecosicyos*, and *Oreosyce*. Analysis of the combined data unexpectedly revealed that a monophyletic *Cucumis *lineage includes an Australian/Asian clade in which cucumber, *C. sativus*, is nested. This then raised the questions about the timing of the Australian connections, which we address with molecular clock dating.

## Results and Discussion

### The non-monophyly of *Cucumis *and why it remained undiscovered; comparison with earlier molecular phylogenies

**Figure 1:**
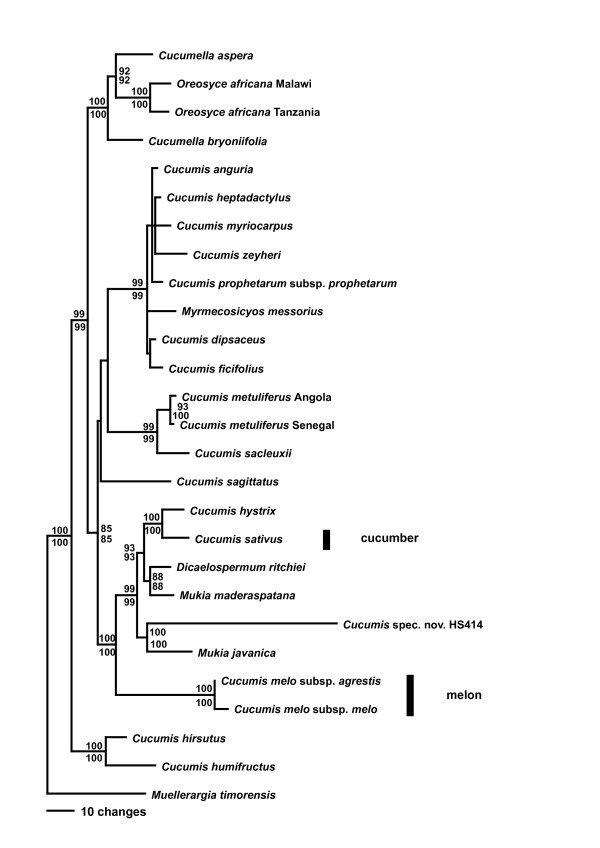
Maximum likelihood tree for *Cucumis *based on combined sequences from chloroplast genes, introns, and a spacer (details see Table 1). The tree is rooted on *Muellerargia*, the closest relative of *Cucumis*, based on the family phylogeny shown in Fig. 4. Parsimony bootstrap values (> 85%) based on 1000 replicates above branches and ML bootstrap values from 100 replicates below branches.

**Figure 2:**
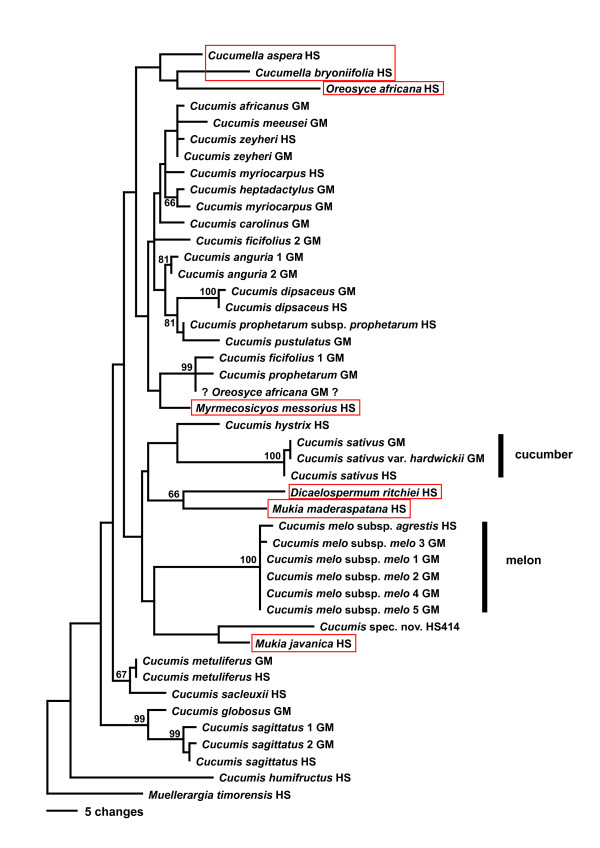
Parsimony tree for *Cucumis *based on sequences from the nuclear internal transcribed spacer, rooted on *Muellerargia *as in Fig. 1. Bootstrap values (> 65%) at branches are based on 1000 replicates. The genera marked with red lines are nested in *Cucumis*, and their species will need to be transferred to make *Cucumis *monophyletic. Species with the letters GM (Garcia-Mas) are from [22], while species labeled HS were generated for this study. The GenBank sequence labeled '?*Oreosyce africana*?' is from misidentified material (see text).

**Figure 3:**
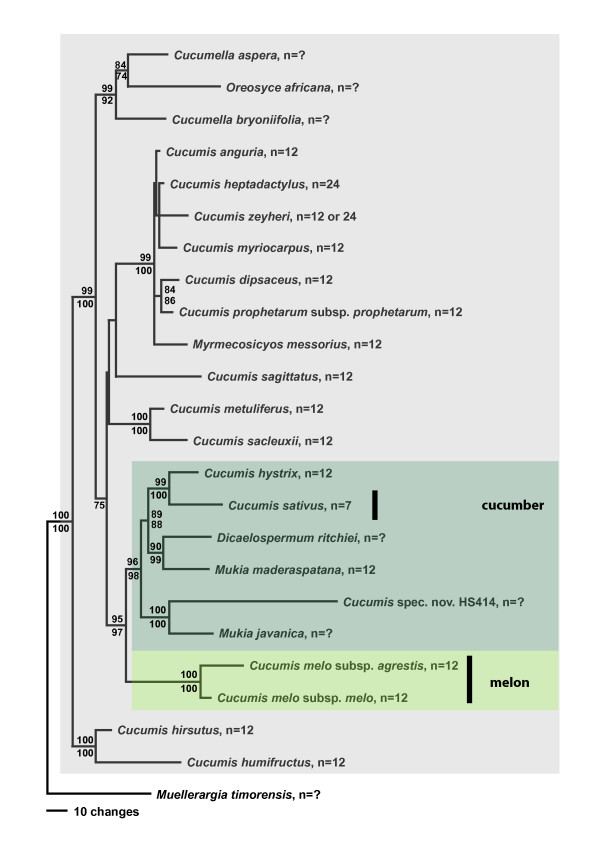
Parsimony tree for *Cucumis *based on the combined chloroplast and nuclear data and rooted on *Muellerargia *as in Fig. 1. Parsimony bootstrap values (> 75%) based on 1000 replicates above branches and ML bootstrap values from 100 replicates below branches. Species on pale grey background occur in Africa (*C. prophetarum *extends into India); the clade marked in grey-green occurs in Australia, the Malaysian region, Indochina, China, and India (*Mukia maderaspatana *extends into the Yemen and sub-Saharan Africa; see Table 1 for geographic ranges); the natural range of melon (*C. melo*) is unclear. Information on chromosome numbers is from the Index to Plant Chromosome Numbers database available online at the Missouri Botanical Garden's web site.

**Figure 4:**
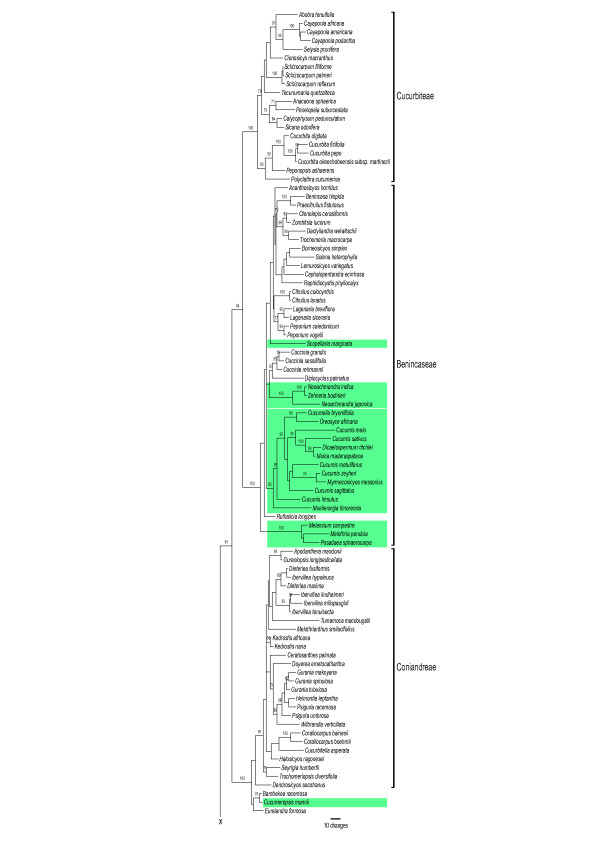
Detail of one of highest global likelihood trees for Cucurbitaceae obtained from combined chloroplast sequences (*matK*, *rbcL*, the *trnL *intron and spacer, and the *rpl20-rps12 *spacer; 4,966 aligned nucleotides; GTR + G), with parsimony bootstrap values based on 100 replicates shown at branches. Modified from 10, which contains the full tree with all 123 genera. Highlighted are the *Cucumis *clade and the genera of Cucumerinae in the most recent morphological classification (11).

Parsimony (MP) and maximum likelihood (ML) analyses of combined sequences from the chloroplast genes *rbcL *and *matK*, the chloroplast intron *trnL*, and the spacers *rpl20-rps12* and *trnL-F*, under the GTR + G + I model yielded a topology (Fig. 1) that was congruent with that obtained from the nuclear internal transcribed spacer region (Fig. 2). Chloroplast and nuclear data were therefore combined, and a parsimony tree from the combined data with MP and ML bootstrap support is shown as Fig. 3 (seven of the species lack ITS sequences, Table 1). In the family-wide analysis (with 123 of 130 genera of Cucurbitaceae sequenced), *Cucumis *is sister to *Muellerargia *(Fig. 4). The genera *Cucumella*, *Dicaelospermum*, *Mukia*, *Myrmecosicyos*, and *Oreosyce *are embedded among species of *Cucumis *(Fig. 3). The remaining genera of Cucumerinae sensu C. Jeffrey [11], *Cucumeropsis *Naudin, *Melancium *Naudin, *Melothria *L., *Posadaea *Cogn., and *Zehneria *Endl. (plus *Neoachmandra *and *Scopellaria *[24]) group far from *Cucumis *(Fig. 4). This fits with their geographic concentration in the New World (where *Cucumis *is absent): *Melancium *is a monotypic genus from Brazil, *Posadaea *a monotypic genus from tropical America, and *Melothria *has ten species in Central America and South America. However, *Cucumeropsis*, with a single species from tropical Africa, and *Zehneria*, *Neoachmandra*, and *Scopellaria*, with 66 species in tropical and subtropical Africa, Madagascar, Asia, New Guinea, and Australia [24] overlap with the natural range of *Cucumis*.

**Table 1:** Species and loci sequenced, their sources and geographic provenience, GenBank accession numbers, and status as nomenclatural types.

Species	DNA source	Geographic origin of the sequenced material	*rbcL *gene	*matK *gene	*trnL *intron	*trnL-F *spacer	*rpl20-rps12 *spacer	*ITS *spacer
*Cucumella aspera *(Cogn.) C. Jeffrey	O. H. Volk 2789 (M)	Namibia	DQ785826	DQ785842	DQ785868	DQ785868	DQ785854	EF091850
*Cucumella bryoniifolia *(Merxm.) C. Jeffrey	M. Wilkins 214b, seeds cult. in Tucson, Arizona	Republic South Africa	DQ535798	DQ536657	DQ536763	DQ536763	DQ648165	EF091851
*Cucumis anguria *L. var. *longaculeata *Kirkbride (section *Aculeatosi*, series *Angurioidei*)	R. Seydel 3439 (M)	Namibia	DQ785827	DQ785843	DQ785869	DQ785869	DQ785855	-
*Cucumis dipsaceus *Spach (section *Aculeatosi*, series *Angurioidei*)	H. Schaefer 05/510 (M)	Dar-Es-Salaam, Tanzania	DQ785828	DQ785844	DQ785870	DQ785870	DQ785856	EF093513
*Cucumis ficifolius *A. Rich. (section *Aculeatosi*, series *Angurioidei*)	J. E. Weiss s.n. (M), cult. BG Munich	Tropical East Africa	DQ785829	DQ785845	DQ785871	DQ785871	DQ785857	-
*Cucumis heptadactylus *Naudin (section *Aculeatosi*, series *Myriocarpi*)	W. Giess 168 (M)	Republic South Africa	DQ785830	DQ785840	DQ785872	DQ785872	DQ785858	-
*Cucumis hirsutus *Sond. (section *Melo*, series *Hirsuti*)	N. B. Zimba et al. 874 (MO)	Zambia	DQ535799	DQ536658	DQ536804	DQ536804	DQ536542	-
*Cucumis humifructus *Stent (section *Melo*, series *Humifructosi*)	H. Merxmüller & W. Giess 30150 (M)	Namibia	DQ785831	DQ785841	DQ785873	DQ785873	DQ785859	EF093514
*Cucumis hystrix *Chak. (subgenus *Cucumis*)	S. Suddee, W. J. J. O. de Wilde & B. E. E. Duyfjes 2503 (L)	Doi Chiang Dao, Thailand	DQ785832	DQ785846	-	DQ785874	DQ785860	EF093515
*Cucumis melo *L. subsp. *melo *(section *Melo*, series *Melo*)	Store-bought cantaloupe	Unknown	DQ535800	DQ536659	DQ536764	DQ536764	DQ648166	-
*Cucumis melo *L. subsp. *agrestis *(Naudin) Pangalo (section *Melo*, series *Melo*)	D. Podlech 32603 (M)	Prov. Nangahar, Afghanistan	DQ785833	DQ785847	DQ785875	DQ785875	DQ785861	EF093516
*Cucumis metuliferus *Naudin (section *Aculeatosi*, series *Metuliferi*)	B. de Winter & W. Marais 4614 (M)	Angola	DQ785834	DQ785848	DQ785876	DQ785876	DQ785862	EF093517
*Cucumis metuliferus *Naudin (section *Aculeatosi*, series *Metuliferi*)	J. Berhaut 7478 (M)	Senegal	DQ785835	DQ785849	DQ785877	DQ785877	DQ785863	-
*Cucumis myriocarpus *E. Mey. ex Naudin (section *Aculeatosi*, series *Myriocarpi*)	S. S. Renner et al. 2801 (M), cult. Mainz BG	Republic South Africa	DQ785836	DQ785850	DQ785878	DQ785878	DQ785864	EF093518
*Cucumis prophetarum *L. subsp.*prophetarum *(section *Aculeatosi*, series *Angurioidei*)	K. H. Rechinger 28768 (M)	Quetta, Pakistan	DQ785837	DQ785851	DQ785879	DQ785879	DQ785865	EF093519
*Cucumis sacleuxii *Paill. & Bois (section *Aculeatosi*, series *Angurioidei*)	H. Schaefer 05/411 (M)	Usambara Mts., Tanzania	DQ785838	DQ785852	DQ785880	DQ785880	DQ785866	EF093520
*Cucumis sagittatus *Peyr. section *Melo*, series *Hirsuti*)	D. Decker-Walters 1124 (FTG)	Namibia	DQ535802	DQ536661	DQ536806	DQ536806	DQ648168	EF093521
*Cucumis sativus *L.; Generic type (subgenus *Cucumis*)	^1 ^S. S. Renner 2745 (M), cult. BG Munich ^2 ^S. S. Renner 2822	^1^Unknown ^2^Guangxi, China	^1^DQ535747	^1^DQ536662	^1^DQ536765	^1^DQ536765	^1^DQ648169	^**2 **^EF093522
*Cucumis *sp. nov. HS414	P. I. Forster 9514 (NE)	Australia	EF174480	EF174478	EF174486	EF174486	EF174482	EF174483
*Cucumis zeyheri *Harvey & Sond. (section *Aculeatosi*, series *Angurioidei*)	D. Decker-Walters 1114 (FTG)	Natal Republic South Africa	DQ535803	DQ536663	DQ536807	DQ536807	DQ648170	EF093523
*Dicaelospermum ritchiei *C.B. Clarke; Generic type; originally spelled *Dicoelospermum*	H. Santapaa 13354 (MO)	Khandala, India	DQ535806	-	DQ536811	DQ536811	DQ536546	EF093524
*Muellerargia timorensis *Cogn. *Zehneria ejecta *F. M. Bailey; Generic type	D. L. Jones 3666 (NE)	Queensland, Australia	DQ535777	DQ536704	DQ536842	DQ536842	DQ536571	EF093525
*Mukia maderaspatana *(L.) M. Roem. The type species of the genus, *M. scabrella *(L.) Wight, is a synonym of this name.	J. Maxwell 02-434 (CMU)	Chiang Mai, Thailand	DQ535761	DQ536705	DQ536843	DQ536843	DQ648182	EF093526
*Mukia javanica *(Miq.) C. Jeffr.	H. Schaefer 05/133 (M)	Yunnan, China	EF174479	EF174477	EF174485	EF174485	EF174481	EF174484
*Myrmecosicyos messorius *C. Jeffr.; Generic type	P. R. O. Bally B15187 (EA)	Lake Elementaita, Kenya	-	DQ536706	DQ535872	-	DQ536572	EF093527
*Oreosyce africana *Hook.f.; Generic type	H. Schaefer 05/450 (M)	Usambara Mts., Tanzania	DQ785839	DQ785853	DQ785881	DQ785881	DQ785867	EF093528
*Oreosyce africana *Hook.f.; Generic type	E. Phillips 2821 (Z)	Malawi	DQ535833	DQ536711	DQ536845	DQ536845	DQ536576	-

Numbers in bold indicate sequences newly generated for this study. Herbarium acronyms follow the Index Herbariorum available online at the New York Botanical Garden's web site. BG = botanical garden. Sections and series of *Cucumis *are from [12].

The sister genus to *Cucumis*, *Muellerargia*, consists of one species in Madagascar and one in Indonesia and Queensland. Both are herbaceous trailers or climbers with straight or apically reflexed anthers and softly spinose fruits. *Muellerargia *has never been recognized as closely related to *Cucumis *[12,25], perhaps because it is extremely poorly collected, with but a few specimens even in major herbaria: The Madagascan species, *Muellerargia jeffreyana *Keraudren, is known from three collections (in the Paris herbarium), and permission was not granted to sacrifice material for this study. It is morphologically similar to the Indonesian-Australian species *M. timorensis *Cogn. [26]. The poor documentation of the genus in herbaria also led to the Australian species being described at least three times; first as *Muellerargia timorensis *Cogn., then as *Melothria subpellucida *Cogn., and then as *Zehneria ejecta *Bailey (syn. *Melothria ejecta *(Bailey) Cogn.).

The *Cucumis *species relationships found here differ from those found in earlier studies [1,20-22]. An unrooted nuclear isozyme tree [21] showed *C. sativus *as the genetically most distant species, while *C. melo *was sister to an African clade. The neighbor-joining tree from nuclear ITS sequences of Garcia-Mas et al. [22] was rooted on *Citrullus lanatus *and *Cucurbita pepo*, and showed *C. sativus *as the first-branching species in the genus, while *C. melo *was sister to a large African clade. Finally, the chloroplast tree of Chung et al. [1] also was rooted on *Citrullus *and showed *C. sativus *and *C. hystrix *as sister to *C. melo *(as did studies focusing on *C. sativus*; e.g., [27]). By contrast, the data presented here (Figs. 1, 2, 3, 4) indicate that (i) the deepest divergence in *Cucumis *is between *C. hirsutus *and *C. humifructus *on the one hand and all other species on the other, (ii) *C. sativus *(cucumber) and *C. hystrix *are closer to *Dicaelospermum *and *Mukia *than they are to any species of *Cucumis*, and (iii) *C. melo *(melon) is sister to a clade comprising *Dicaelospermum*, *Mukia, C. sativus*, *C. hystrix*, and a new species from Australia (HS414).

There are several possible explanations for the contrasting findings of the earlier phylogenetic studies. First, the use of distant outgroups might have "attracted" the long-branched (i.e., mutation-rich) *C. sativus*, pulling it to the base of the tree. Garcia-Mas et al. [22] and Chung et al. [1] used *Citrullus lanatus *and/or *Cucurbita pepo *as sole outgroups. Both taxa are many clades, and millions of years of evolution, removed from the *Cucumis *clade (Fig. 4) and therefore add long branches to neighbor-joining and parsimony analyses [1,22]. The inclusion of these long branches could have caused long-branch attraction between them and *C. sativus*.

A second reason why previous molecular phylogenetic studies were unable to test the monophyly of *Cucumis *and to infer the sister clades of cucumber and melon is that they did not include a sufficiently broad sample of taxa. For example, rigidly testing the monophyly of *Cucumis *section *Melo *required sequencing all of its species, *C. melo*, *C. hirsutus*, *C. humifructus*, and *C. sagittatus*. Results (Figs. 1, 2, 3) show that *C. hirsutus *and *C. humifructus*, rather than being close to *C. melo*, are sister to all other species of *Cucumis *sensu lato, that is, including all five genera nested in *Cucumis*.

Another possible reason for apparent differences between earlier topologies and the phylogeny found here is insufficient signal in the data and misidentified material. Comparison of the ITS sequences of Garcia-Mas et al. [22] to our ITS sequences showed that the sequence labeled *Cucumis membranifolius *in GenBank (AJ488223) and *Oreosyce africana *in the published paper (these names refer to the same species fide [12]), does not represent *Oreosyce africana*. The sequence came from a seed provided by the North Central Regional Plant Introduction Station in Ames, Iowa, and since there is no voucher, its identification cannot be verified. We also could not reproduce the topology and bootstrap support obtained in the original paper [22], partly probably because the phylogenetic signal in the data is weak, resulting in many equally likely trees. Garcia-Mas et al. [22] included sequences resulting from direct sequencing as well as sequences obtained by pGEM-T Easy Vector cloning and found sequences from multiple accessions generally grouping by species. Our *Cucumis *ITS sequencing confirmed these authors' assessment that ITS lineage sorting is not a problem in *Cucumis*. The two *C. ficifolius *sequences obtained by Garcia-Mas et al. [22] that do not group (Fig. 2) come from different plants and may simply represent different species; however, since the material is unvouchered, the identifications cannot be checked.

### Implications for the evolution and biogeography of *Cucumis*

The phylogeny from the combined nuclear and chloroplast data (Fig. 3) implies that the deepest divergence lies between the common ancestor of *C. hirsutus *and *C. humifructus *and the stem lineage of the remainder of the genus. From the geographic ranges of the species of *Cucumis sensu lato *(i.e., the natural clade identified here) and its sister genus *Muellerargia *(with one species in tropical Australia and Indonesia, the other in Madagascar), the area where *Cucumis *may have originated cannot reliably be inferred. Strict and semi-parametric molecular clocks indicated that the deepest divergence in *Cucumis *may date back to 48–45 my and that the split between the *C. melo *lineage and its Australian/Asian sister clade is only slightly younger. The divergence of *C. sativus *from *C. hystrix *may be about 8 my old and that of their common ancestor from the ancestor of *Dicaelospermum *and *Mukia maderaspatana *about 19 my. The bulk of the African species appears to have evolved more recently. The absence of a fossil constraint within *Cucumis*, however, cautions against over-confidence in the molecular clock estimates.

Based on the tree (Fig. 3), the earliest divergence events in *Cucumis *likely took place in Africa. However, contrary to the traditional classification [12], which groups *C. melo *with the African *C. hirsutus*, *C. humifructus*, and *C. sagittatus*, melon is closest to an Australian/Asian clade (marked in grey-green in Fig. 3) that comprises an undescribed Australian species [28], species currently placed in *Mukia *(*M. javanica*, *M. maderaspatana*), *Dicaelospermum ritchiei *from Western India (recently transferred to *Mukia *[29]), and *Cucumis sativus *and *C. hystrix *from India, China, Burma, and Thailand. In addition to the two species we sequenced, *Mukia *comprises three others [29], its overall geographic range extending from Indo-China southeast to Java, Borneo, and the Philippines, and west through India, Pakistan, and the Yemen into sub-Saharan Africa. Given the geographic distribution of its extant closest relatives (Fig. 3), *C. melo *itself could have originated somewhere in Asia and then reached Africa from there, rather than originating in Africa as traditionally assumed [14,15]. Notably, Indian melon landraces exhibit the largest isozyme variation among Asian melons [16] and Australia is a center of complex morphological variation of *C. melo *[28].

The evolution of morphological traits relevant for *Cucumis *breeders, for example fruit type, habit, and sexual system, will need to be reinterpreted based on the phylogenetic relationships presented here. Most of the 52 described species in the *Cucumis *clade are monoecious perennials, and the monoecious sexual system and perennial habit may be the ancestral condition from which an annual habit and dioecy appear to have evolved several times. However, the sexual system and habit of key taxa, such as *Dicaelospermum, Muellerargia*, and the as yet undescribed species from Australia [28] (Fig. 3) are not reliably known because species are under-collected and have not been studied in the field. Of the 17 species of *Cucumis *not yet sequenced, most are monoecious perennials; only *C. kalahariensis *A. Meeuse and *C. rigidus *Sond. are dioecious and perennial. Species currently placed in *Mukia *[29] and *Cucumella *[13] are mostly monoecious and perennial. The evolution of smooth fruits from spiny fruits, a traditional key character in *Cucumis*, and the mode of fruit opening are much more plastic than formerly thought. For example, in *Oreosyce africana *and *Muellerargia timorensis *the fruits open explosively [[30]; I. Telford, Beadle Herbarium, Armidale, personal communication, Feb. 2007); in *C. humifructus*, fruits mature below ground and are then dug up and the seeds dispersed by antbears, *Orycteropus afer *[31]; in the new species from Australia (HS414 in Figs. 1 and 2), the developing fruit is pushed into rock crevices by the elongating pedicel and also matures below ground; and in *Myrmecosicyos messorius *the fruits are tiny and apparently dispersed by harvesting ants around whose nest entrances the species grows.

## Conclusion

Based on congruent nuclear and chloroplast phylogenies we conclude that a monophyletic *Cucumis *comprises an old Australian/Asian clade that includes cucumber and at least eight other species, most of them currently placed in *Mukia*. The new insights about the closest relatives of melon and cucumber have implications for ongoing genomics efforts. It is known that *Cucumis *organellar genomes are unusually labile. Thus, in *C. sativus*, *rbcL *has been transferred from the plastome to the mitochondrial genome [4], and huge amounts of degenerate repetitive DNA have accumulated in *C. sativus *mitochondria [5-7]. The seven meiotic chromosomes of *C. sativus *are larger than the 12 of its wild sister species or progenitor *C. hystrix *[32] and consist of six metacentrics and one submetacentric chromosome [33]. To infer the genome rearrangements that must have taken place during the evolution and domestication of *C. sativus*, analyses of co-linearity will be required between the cucumber lineage and its closest relatives *Dicaelospermum ritchiei *and species of *Mukia*. Finally, the possibility that *C. melo *may have evolved in Asia and reached Africa secondarily needs to be tested.

## Methods

### Taxon Sampling and Data Sets Analyzed

Table 1 lists all species sampled with authors, status as generic types where applicable, plant sources, and GenBank accession numbers [TreeBASE: study accession S1604, matrix accession M2887, M3250 and M3251]; 79 chloroplast and 20 ITS sequences were newly generated for this study. Species concepts and generic assignments throughout this study follow recent classifications [11-13,29], although as a result of this study, species in several genera have been transferred into *Cucumis *([34]; this also provides a morphological key to the 52 described species). To resolve species relationships within *Cucumis*, we added sequences from the nuclear internal transcribed spacer region (220 nt of ITS 1, 163 nt of the 5.8S gene, and 240 nt of ITS 2) for the same species for which chloroplast data were generated. DNA extraction, purification, and sequencing of the selected loci followed standard procedures [10]. All PCR products were sequenced in both directions. Direct PCR amplification of ITS yielded single bands and unambiguous base calls, except in *C. ficifolia*, the sequences of which were therefore not used. Sequences were edited and assembled with the Sequencher software (Gene Codes) and aligned by eye, using MacClade [35]. The aligned chloroplast matrix comprised 4,375 positions after exclusion of a poly-T run in the *matK *gene, a poly-A run in the *trnL *intron, a TATATA microsatellite region in the *trnL-F *intergenic spacer and a poly-A run in the *rpl20-rps12 *intergenic spacer. The aligned ITS matrix comprised 677 aligned positions, and we excluded a poly-G stretch of 25 nt and a poly-C stretch of 17 nt from the ITS1 and a poly-C stretch of 11 nt from ITS2.

### Phylogenetic Analyses

Equally weighted parsimony analyses were conducted using PAUP 4.0b10 [36]. The search strategy involved 100 random taxon addition replicates with tree-bisection-reconnection branch swapping, MulTrees and Steepest Descent in effect, no limit on trees in memory, and saving all optimal trees. For MP analyses, gaps were treated as missing data, while for ML searches (below) they were mostly removed. To assess node support, parsimony bootstrap analyses were performed using 1000 replicate heuristic searches, each with 10 random addition replicates and otherwise the same settings as used for tree searches. More computationally intensive heuristic approaches have been found not to increase the reliability of bootstrapping [37]. Maximum likelihood analyses and bootstrapping were performed using GARLI 0.951 [38]. GARLI searches relied on the GTR + G + P-invar model, which ModelTest 3.06 [39] selected as the best fitting model for the combined data. Parameters were estimated over the duration of specified runs.

### Molecular clock dating

Molecular clock dating in Cucurbitaceae is problematic because of the family's scarce fossil record. Without multiple calibrations, such as could come from several securely assigned fossils, relaxed molecular clock methods have been shown to perform poorly [40-42]. We therefore relied on a strict clock approach and compared it with results obtained with the semi-parametric penalized likelihood approach [40] implemented in r8s vs. 1.7). For strict clock dating, we employed the maximum likelihood topology obtained (under GTR + G) with the family data set of Kocyan et al. [10] augmented by the *Cucumis *sequences generated for this study for a total of 193 taxa and 5,028 aligned nucleotide positions. The tree was imported into PAUP [36], rooted on Corynocarpaceae, and *rbcL *branch lengths were then calculated under a GTR + G + I + strict clock model. Branch lengths were saved and a mutation rate obtained by dividing the distance from the most recent common ancestor (mrca) of *Trichosanthes *to the present (0.01416) by 65 my, based on the oldest seeds assigned to this genus [43]. Using the resulting rate of 0.000218 substitutions/site/my, we obtained an age of 47.6 my for the mrca of *Cucumis *by dividing the distance from the basal divergence in *Cucumis *to the present (0.01037) by 0.000218. The time of the divergence of *C. melo *from its sister clade was calculated accordingly (0.00975 : 0.000218 = 44.7 my). To check this strict clock estimate based on *rbcL*, we imported the 193-taxon-ML tree with branch lengths from the combined chloroplast data (5,028 nt) into r8s and ran a cross validation analysis, using the following upper and lower temporal constraints. The mrca of the family Cucurbitaceae was constrained to maximally 100 my and minimally 65 my old based on Cucurbitales family relationships and fossil records [9,43]; the mrca of *Trichosanthes *was constrained to minimally 65 my [42]; and the mrca of an endemic clade of two species occurring on Hispaniola was constrained to maximally 30 my old based on the oldest ages of Dominican amber [44]. Penalized likelihood yielded an age of 44.9 my for the mrca of *Cucumis*.

## Abbreviations

ML, maximum likelihood; MP, maximum parsimony; mrca, most recent common ancestor; my, million years; nt, nucleotide.

## Authors' contributions

SR, HS, and AK obtained the material, AK organized the sequencing and alignments for non-*Cucumis *Cucurbitaceae, HS did the sequencing and alignments for this study, and SR designed the project, performed molecular clock analyses, and wrote the manuscript. All authors ran tree searches, and all read and approved the final submission.

## References

[B1] Chung S-M Staub JE Chen J-F Molecular phylogeny of *Cucumis *species as revealed by consensus chloroplast SSR marker length and sequence variation Genome 2006 49 219 229 10.1139/G05-101 16604104

[B2] Pitrat M Chauvet M Foury C Diversity, history, and production of cultivated cucurbits Acta Hort 1999 492 21 28

[B3] Kim JS Jung JD Lee JA Park HW Oh KH Jeong WJ Choi DW Liu JR Cho KY Complete sequence and organization of the cucumber (*Cucumis sativus *L. cv. Baekmibaekdadagi) chloroplast genome Plant Cell Rep 2006 25 334 340 10.1007/s00299-005-0097-y 16362300

[B4] Cummings MP Nugent JM Olmstead RG Palmer JD Phylogenetic analysis reveals five independent transfers of the chloroplast gene *rbcL *to the mitochondrial genome in angiosperms Curr Genet 2003 43 131 138 12695853 10.1007/s00294-003-0378-3

[B5] Bendich AJ Anderson RS Novel properties of satellite DNA from muskmelon Proc Natl Acad Sci USA 1974 71 1511 1515 4524654 10.1073/pnas.71.4.1511 PMC388260

[B6] Ward BL Anderson RS Bendich AJ The mitochondrial genome is large and variable in a family of plants (Cucurbitaceae) Cell 1981 25 793 803 10.1016/0092-8674(81)90187-2 6269758

[B7] Lilly JW Havey MJ Small, repetitive DNAs contribute significantly to the expanded mitochondrial genome of cucumber Genetics 2001 159 317 328 11560907 10.1093/genetics/159.1.317 PMC1461790

[B8] Ritschel PS de Lima Lins TC Tristan RL Cortopassi-Buso GS Amauri-Buso J Ferreira ME Development of microsatellite markers from an enriched genomic library for genetic analysis of melon (*Cucumis melo *L.) BMC Plant Biology 2004 4 9 15149552 10.1186/1471-2229-4-9 PMC419974

[B9] Zhang L-B Simmons MP Kocyan A Renner SS Phylogeny of the Cucurbitales based on DNA sequences of nine loci from three genomes: implications for morphological and sexual system evolution Mol Phyl Evol 2006 39 305 322 10.1016/j.ympev.2005.10.002 16293423

[B10] Kocyan A Zhang L-B Schaefer H Renner SS A multi-locus chloroplast phylogeny for the Cucurbitaceae and its implications for character evolution and classification Mol Phyl Evol 2007 in press 10.1016/j.ympev.2006.12.022 17321763

[B11] Jeffrey C A new system of Cucurbitaceae Bot Zhurn 2005 90 332 335

[B12] Kirkbride JH Jr Biosystematic monograph of the genus Cucumis (Cucurbitaceae) 1993 Boone, NC: Parkway Publishers

[B13] Kirkbride JH Jr Revision of *Cucumella *(Cucurbitaceae, Cucurbitoideae, Melothrieae, Cucumerinae) Brittonia 1994 46 161 186 10.2307/2807230

[B14] Whitaker TW Davis GN Cucurbits – Botany, Cultivation, Utilization 1996 New York: Interscience Publ

[B15] Robinson RW Decker-Walters DS Cucurbits. (Crop Production Science in Horticulture no. 6) 1997 New York: Cab International

[B16] Akashi Y Fukuda N Wako T Masuda M Kato K Genetic variation and phylogenetic relationships in East and South Asian melons, *Cucumis melo *L., based on the analysis of five isozymes Euphytica 2002 125 385 396 10.1023/A:1016086206423

[B17] Linnaeus C Species plantarum 1735 1 Stockholm, Impensis Laurentii Salvii

[B18] Jeffrey C Notes on Cucurbitaceae, including a proposed new classification of the family Kew Bull 1962 15 337 371

[B19] Chen J-F Staub JE Tashiro Y Isshiki S Miyazaki S Successful interspecific hybridization between *Cucumis sativus *L. and *C. hystrix *Chakr Euphytica 1997 96 413 419 10.1023/A:1003017702385

[B20] Perl-Treves R Galun E The *Cucumis *plastome: physical map, intrageneric variation and phylogenetic relationships Theor Appl Genet 1985 71 417 429 10.1007/BF00251182 24247447

[B21] Perl-Treves R Zamir D Navot N Galun E Phylogeny of *Cucumis *based on isozyme variability and its comparison with plastome phylogeny Theor Appl Genet 1985 71 430 436 10.1007/BF00251183 24247448

[B22] Garcia-Mas J Monforte AJ Arús P Phylogenetic relationships among *Cucumis *species based on the ribosomal internal transcribed spacer sequence and microsatellite markers Plant Syst Evol 2004 248 191 203 10.1007/s00606-004-0170-y

[B23] Jeffrey C A review of the Cucurbitaceae Bot J Linn Soc 1980 81 233 247

[B24] De Wilde WJJO Duyfjes BEE Redefinition of *Zehneria *and four new related genera (Cucurbitaceae), with an enumeration of the Australasian and Pacific species Blumea 2006 51 1 88

[B25] Müller EGO Pax F Engler A, Prantl K Cucurbitaceae Die natürlichen Pflanzenfamilien nebst ihren Gattungen und wichtigeren Arten insbesondere den Nutzpflanzen 1889 IV, 5 34 Leipzig: W. Engelmann 1 39

[B26] Keraudren M Présence du genre indonésien *Muellerargia *(Cucurbitaceae) a Madagascar Adansonia 1965 5 421 424

[B27] Zhuang F-Y Chen J-F Staub JE Qian C-T Taxonomic relationships of a rare *Cucumis *species (*C. hystrix *Chakr.) and its interspecific hybrid with cucumber Hortscience 2006 41 571 574

[B28] Telford IR Cucurbitaceae. Fl. Australia 8 1982 205 158 198

[B29] De Wilde WJJO Duyfjes BEE *Mukia *Arn. (Cucurbitaceae) in Asia, in particular in Thailand Thai Forest Bull (Bot.) 2006 34 38 52

[B30] Jeffrey C Milne-Redhead, Polhill RM *Cucurbitaceae* Flora of Tropical East Africa 1967 London: Royal Botanic Gardens, Kew

[B31] Meeuse AJD A possible case of interdependence between a mammal and a higher plant Arch Néerl Zool 1958 13 Suppl 314 318

[B32] Chen J-F Luo X-D Qian C-T Jahn MM Staub JE Zhuang F-Y Lou Q-F Ren G *Cucumis *monosomic alien addition lines: morphological, cytological, and genotypic analyses Theor Appl Genet 2004 108 1343 1348 10.1007/s00122-003-1546-z 14666371

[B33] Koo DH Choi HW Cho J Hur Y Bang JW A high-resolution karyotype of cucumber (*Cucumis sativus *L. 'Winter Long') revealed by C-banding, pachytene analysis, and RAPD-aided fluorescence in situ hybridization Genome 2005 48 534 540 10.1139/g04-128 16121249

[B34] Schaefer H *Cucumis *(Cucurbitaceae) must include *Cucumella*, *Dicoelospermum*, *Mukia*, *Myrmecosicyos*, and *Oreosyce: *a recircumscription based on nuclear and plastid DNA data Blumea 2007 in press

[B35] Maddison DR Maddison WP *MacClade*, version 4.05 2003 Sunderland, MA: Sinauer Associates

[B36] Swofford DL PAUP*: Phylogenetic Analysis Using Parsimony (*and other methods) 2002 Sunderland, MA: Sinauer Associates

[B37] Müller K The efficiency of different search strategies in estimating parsimony jackknife, bootstrap, and Bremer support BMC Evol Biol 2005 5 58 16255783 10.1186/1471-2148-5-58 PMC1282575

[B38] Zwickl DJ Genetic algorithm approaches for the phylogenetic analysis of large biological sequence datasets under the maximum likelihood criterion 2006. Ph.D. dissertation, The University of Texas at Austin

[B39] Posada D Crandall KA Modeltest: testing the model of DNA substitution Bioinformatics 1998 14 817 818 10.1093/bioinformatics/14.9.817 9918953

[B40] Sanderson MJ Estimating absolute rates of molecular evolution and divergence times: a penalized likelihood approach Mol Biol Evol 2002 19 101 109 11752195 10.1093/oxfordjournals.molbev.a003974

[B41] Pérez-Losada M Høeg JT Crandall KA Unraveling the evolutionary radiation of the Thoracican barnacles using molecular and morphological evidence: a comparison of several divergence time estimation approaches Syst Biol 2004 53 244 264 10.1080/10635150490423458 15205051

[B42] Ho SY Phillips MJ Drummond AJ Cooper A Accuracy of rate estimation using relaxed-clock models with a critical focus on the early metazoan radiation Mol Biol Evol 2005 22 1355 1363 10.1093/molbev/msi125 15758207

[B43] Collinson ME Boulter MC Holmes PR Benton MJ Magnoliophyta (Angiospermae) The Fossil Record 2 1993 Chapman and Hall, London 809 841, 864

[B44] Iturralde-Vinent MA MacPhee RDE Age and paleogeographical origin of Dominican amber Science 1996 273 1850 1852 10.1126/science.273.5283.1850

